# A pharmacist’s guide to human papillomavirus (HPV)

**DOI:** 10.1177/17151635221116342

**Published:** 2022-09-02

**Authors:** Cheryl E. Cable, Cert Prosth, Maxillofacial Prosth, Ian Vandermeer, Adrian Poon, Ross T. Tsuyuki

**Affiliations:** Faculty of Medicine and Dentistry, University of Alberta, Edmonton, Alberta; Faculty of Medicine and Dentistry, University of Alberta, Edmonton, Alberta; Faculty of Medicine and Dentistry, the University of Waterloo, Kitchener, Ontario; Faculty of Medicine and Dentistry, University of Alberta, Edmonton, Alberta

## HPV and cancers

**Figure 1:**
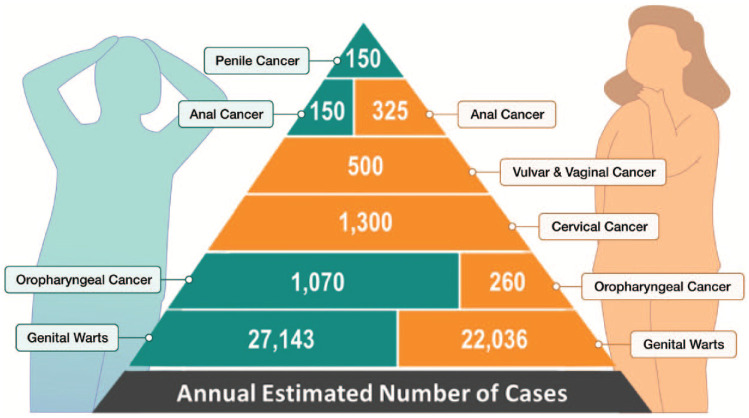
Canadian human papillomavirus–associated cancers statistics Canadian Cancer Statistics 2016.

Human papillomavirus (HPV) is a common infection, affecting up to 75% of sexually active people.^1^ Importantly, HPV infection is known to cause cancer (Figure 1).

We often think of HPV mainly as a major cause of cervical cancer. In fact, oropharyngeal cancers outnumber cases of cervical cancer due to HPV.^2^ The incidence rate for oropharyngeal cancers has been steadily increasing since the mid-1990s, particularly in men. It has been increasing at a rate of 4.5 times faster in males than in females.^3^

These cancers are *preventable* with vaccination, but already low vaccination rates have been made worse by the pandemic. For example, Ontario school-based HPV vaccination rates have dropped to as low as 5.2% and 0.8% for the 2019-2020 and 2020-2021 school years.^4^

## What can you do as a pharmacist today?

### Identify

Identify patients who would benefit from HPV vaccination. The Government of Canada’s *Human Papillomavirus Vaccine: Canadian Immunization Guide*^5^ indicates the vaccine should be used for anyone over the age of 9, immunocompromised or with multiple sexual partners that increase virus exposure. The National Advisory Committee on Immunization and the Canadian Immunization Guide have a table indicating the “Recommended Immunization Schedule and HPV Vaccine, by Group.” If there is any doubt about who to include for vaccination, this reference can help answer questions.^5^

You can set up case-finding strategies to proactively identify patients meeting these criteria. Set up an HPV campaign in your pharmacy. This could also include establishing collaborative relationships with dental professionals—they are already screening for oral cancers and will be writing prescriptions for HPV vaccination. Expect prescriptions for vaccines and questions for further education from dental offices.

Incorporate a vaccination history into your regular activities, such as every medication history, new patient assessment and all medication reviews that you do.

### Inform

Inform patients of the benefits of HPV vaccination. Ask “Are you aware of your risk of certain cancers from HPV infection?” Mention other preventive measures: vaccines, smoking cessation, reducing alcohol consumption, physical barriers such as condoms and limiting the number of sexual partners.

Have handouts on HPV for distribution to your patients (e.g., https://www.gardasil9.ca).

### Vaccinate

Be there to vaccinate your patients. Make sure they get their second dose. Support walk-ins from dental offices. Understand your patients’ insurance coverage for HPV.

## Conclusion

While the COVID-19 pandemic has highlighted pharmacists as immunization superheroes, it has also caused a major reduction in other kinds of vaccination. By proactively case-finding for HPV vaccine candidates, incorporating vaccination histories into your assessments/medication reviews and partnering with dental professionals, pharmacists can also become cancer prevention superheroes. Please see the accompanying infographic on HPV and the pharmacists' role, packaged with this journal (also available at www.cpjournal.ca). ■
